# MiR-552-3p Regulates Multiple Fibrotic and Inflammatory genes Concurrently in Hepatic Stellate Cells Improving NASH-associated Phenotypes

**DOI:** 10.7150/ijbs.80760

**Published:** 2023-07-03

**Authors:** Ningning Ma, Aijun Hou, Xiangyu Pan, Fuguang Sun, Xiaoding Xu, Chuwei Yu, Rongtao Lai, Ruimin Huang, Likun Gong, Qing Xie, Jing Chen, Jin Ren

**Affiliations:** 1 Center for Drug Safety Evaluation and Research, State Key Laboratory of Drug Research, Shanghai Institute of Materia Medica, Chinese Academy of Sciences, 501 Haike Road, Shanghai 201203, China.; 2 School of Life Science and Technology, ShanghaiTech University, 100 Haike Road, Shanghai 201210, China.; 3 University of Chinese Academy of Sciences, No.19A Yuquan Road Beijing 100049, China.; 4 Department of Pharmaceutics, College of Pharmaceutical Sciences, Soochow University, Suzhou 215123, China.; 5 School of Chinese Materia Medica, Nanjing University of Chinese Medicine, Nanjing 210023, China.; 6 Department of Infectious Diseases, Ruijin Hospital, Shanghai Jiaotong University School of Medicine, 197 Ruijin 2nd Road, Shanghai 200025, China.

**Keywords:** MiR-552-3p, NASH, Fibrosis, Inflammation, Synergistic multi-target suppression

## Abstract

Non-alcoholic steatohepatitis (NASH) is a chronic liver disease characterized by hepatic steatosis, inflammation, and progressive fibrosis. Our previous study demonstrated that microRNA-552-3p (miR-552-3p) was down-regulated in the livers of patients with NASH and alleviated hepatic glycolipid metabolic disorders. However, whether miR-552-3p affects NASH progression remains unclear. In this current study, we found that hepatic miR-552-3p expression was negatively correlated with the degree of liver fibrosis and inflammation of NASH patients. Interestingly, the level of miR-552-3p was decreased during hepatic stellate cell (HSC) activation *in vitro*. Overexpression of miR-552-3p could not only inhibit the expression of fibrotic and inflammatory genes, but also restrain the activation of TGF-β1/Smad3 signaling pathway by down-regulating the expression of *TGFBR2* and *SMAD3* in HSCs, finally suppressing HSC activation. More importantly, overexpression of miR-552-3p ameliorated liver fibrosis and inflammation in two murine models: high fat/high fructose/high cholesterol diet-induced NASH model and carbon tetrachloride (CCl_4_)-treated liver fibrosis model. In conclusion, miR-552-3p plays a crucial role in the pathogenesis of NASH by limiting multiple fibrotic and inflammatory pathways in HSCs, which may shed light on its therapeutic potential in NASH.

## Introduction

Non-alcoholic fatty liver disease (NAFLD), renamed metabolic-associated fatty liver disease (MAFLD), is a prevalent chronic disease worldwide affecting over 30% of the general populations according to the statistics by epidemiological surveys^1^. The spectrum of MAFLD ranges from simple steatosis to more severe stages of liver injury including non-alcoholic steatohepatitis (NASH), advanced fibrosis, cirrhosis and hepatocellular carcinoma (HCC)^2^. As a serious spectrum featured by liver inflammation and/or different degrees of fibrosis, NASH will lead to a significant health burden with no approved therapy currently^3^. Therefore, the resolution of inflammation and reversal of fibrosis are two essential end points in the ongoing clinical trials. However, due to the involvement of multiple cytokines and stimuli in the pathogenesis of NASH, the exact mechanisms by which inflammation and fibrosis are induced and regulated remain largely unknown.

Hepatic stellate cells (HSCs) are the key effector cells in liver fibrosis by producing inflammatory and fibrotic mediators that lead to extracellular matrix (ECM) deposition^4^. They maintain quiescent phenotype under physiological conditions and are activated by the crosstalk among inflammation, growth factors, nuclear receptor signaling, ECM interactions and metabolic signals^5^. As a primary driver of HSCs activation, the transforming growth factor β1 (TGF-β1) binds to TGF-β receptors, leading to phosphorylation of SMAD family member 2/3 (SMAD2/3) which shuttles into the nucleus after forming the complex with SMAD4 to promote the expression of hepatic fibrotic genes^6^. Besides the collagen production, the function of activated HSCs has also been discovered in regulating the immune reactions of injured liver, which also contributes to aggravating hepatic inflammation in NASH. Upon activation, HSCs can secrete proinflammatory cytokines, including TGF-β, CCL2, etc. to recruit lymphocytes^7^. In view of the key role of HSCs in the pathogenesis of NASH, it is necessary to investigate in depth the underlying mechanism of HSC activation.

MicroRNAs (miRNAs) are a class of small non-coding RNA molecules that can mediate metabolic homeostasis by transcriptional and post-transcriptional regulatory mechanisms^8^. To date, multiple miRNAs have been discovered to be involved in regulating the progression of NASH, such as miR-223, miR-29, miR-124, miR-378, etc.^9^-^11^. For example, miR-378 that is regulated by Smo-dependent activation of p65 can inhibit Gli3 expression to limit activation of HSC, thereby reducing hepatic damage^12^. MiR-124 directly targets IQGAP1 which can promote the secretion of inflammatory cytokines in TNF-α-induced LX-2 cells^13^. These findings suggest that the changes of miRNAs in stress-responsive HSCs may be involved in the regulation of liver fibrotic and inflammatory responses. We have ever reported that miR-552-3p was able to modulate hepatic glycolipid metabolism and its contents in the livers of NASH patients was negatively correlated with hepatic lipid accumulation^14^. Unexpectedly, the clinical data also showed that the content of miR-552-3p had a more significant negative correlation with the degree of liver inflammation and fibrosis. Given this finding, we speculate that miR-552-3p might have a regulatory role in the fibrosis and inflammation during the progression of NASH.

In the current study, we demonstrated that the level of miR-552-3p in LX-2 cells was decreased in response to several HSC activators, which did not occur in monocyte cell line THP-1 cells. Therefore, we further investigated the effects of miR-552-3p on fibrosis and inflammation in LX-2 cells and two mouse models: high fat/high fructose/high cholesterol diet-induced NASH model and carbon tetrachloride (CCl_4_)-treated liver fibrosis model. The *in vitro* and *in vivo* experiments suggested that miR-552-3p inhibited multiple fibrotic and inflammatory genes, and down-regulated the TGF-β1/Smad3 signaling pathway via inhibiting SMAD3 and TGFBR2, thus ameliorating liver fibrosis and inflammation. To sum up, our results suggested that miR-552-3p might participate in the pathogenesis of NASH and play a protective role in ameliorating NASH-associated inflammation and fibrosis.

## Materials and Methods

### Reagents and antibodies

The phorbol 12-myristate 13-acetate (PMA) was purchased from CSNpharm (China, CSN11701) and dissolved in DMSO. The LPS (Sigma-Aldrich, USA, L2630), TGF-β1 (SinoBiological, China, 10804-HNAC) and CCL2 (GenScript, China, Z03292) were dissolved in H_2_O. The Z-guggulsterone/GS (Selleck, Houston, S6812), GW9662 (Selleck, Houston, S2915), GW4064, Rosiglitazone/RSG and GW3965 (MCE, China, HY-50108, HY-17386 and HY-10627A) were dissolved in DMSO. The antibodies used were as follows: Collagen I and p-Smad3 (abcam, USA, ab260043, ab52903), t-Smad3 and GAPDH (Cell Signaling Technology, USA, 9523, 5174), α-SMA (Proteintech, 23081-1-AP).

### Clinical subjects

All the subjects were recruited from Ruijin Hospital (Shanghai, China). A total of 12 liver samples were collected from NASH patients by performing liver biopsy. All samples were flash-frozen in liquid nitrogen, and stored at -80°C until subsequent molecular analysis. The clinical characteristics of every patient has been exposed before^14^.

### Cell culture and treatment

The LX-2 and THP-1 cells were cultivated in RPMI-1640 medium containing 10% fetal bovine serum (FBS) and 1% penicillin/streptomycin (P/S). HEK293 cells were cultured in Dulbecco's Modified Eagle Medium containing 10% FBS and 1% P/S. All cells were cultured in a humidified atmosphere with 5% CO2 at 37°C. THP-1 cells were differentiated using 160 nM PMA for 48 h. The LX-2 and THP-1 cells were treated with TGF‐β1(10 ng/ml) or CCL2(50 ng/ml) for 24 h, or with LPS (800 ng/ml) for 8h in serum-free medium. The LX-2 cells were treated with Z-guggulsterone/GS (10 μM), GW4064 (2 μM), GW3965 (2 μM), GW9662 (1 μM), RSG (5 μM) or DMSO for 24 h in RPMI-1640 medium after the cells were transfected with negative control (NC) or miR-552-3p for 24 h.

### MiRNA mimics, Small interfering RNA and plasmid transfection

The miR‐552-3p mimics, mutant mimics (miR-552-3p-5mut, miR-552-3p-3mut, miR-552-3p-tmut) and NC, Anti-miR-552-3p and Anti-NC, the IPO8 siRNA (si‐IPO8) and si‐NC were transfected into LX-2 cells separately using Lipofectamine RNAimax. The constructed psiCHECK2-COL3A1/MMP-2/CCL2/SMAD3/TGFBR2 vectors were transfected into HEK293 cells using Lipofectamine 2000. The mimics and siRNAs sequences as listed in Table 1 and Table S1 were chemically synthesized by GenePharma (Shanghai, China).

### Real‐time qPCR (RT‐qPCR)

Total RNA was extracted from cells or liver homogenates using Trizol reagent (Takara, Japan) according to the manufacturer's protocol. Total RNA was reverse-transcribed using the PrimeScript RT Master Mix (Takara), followed by qPCR with Hieff® qPCR SYBR Green Master Mix (Yeasen, China) and analyzed on 7500 Fast Real-Time PCR System (Thermo Fisher Scientific, USA). The relative mRNA level was calculated by the 2^(-ΔΔCt)^ method with GAPDH as an internal control. For the quantification of miR-552-3p, total RNA was reversely transcribed and amplified using TaqMan MicroRNA assay kit (Invitrogen, USA). U6 snRNA was used as an internal loading control. The primer sequences were listed in Table S2.

### Western blotting

Western blotting was used to measure the protein expression levels of Collagen I, p-Smad3, t-Smad3 and α-SMA both in LX-2 cells and mouse liver tissues. Protein levels were detected by using the HRP-conjugated secondary antibody and peroxidase-catalyzed chemiluminescence.

### Cell viability determination

CCK-8 assay was used to measure LX-2 cell viability. Cells were plated at 5×10^3^ cells per well in 96-well plates for 48, 72, 96, 120 h after transfected with mimics. Then, the cells were incubated with 100 µL RPMI-1640 with 10 µL CCK-8 for another 1 h at 37°C. The resulting product was measured at 450 nm using a microplate reader.

### Dual‐luciferase reporter assay

A dual luciferase reporter assay was employed to examine the interaction between five genes (*COL3A1, MMP-2, CCL2, SMAD3, TGFBR2*) and miR-552-3p according to a previously reported method. The 3'-UTRs from their mRNAs were amplified and inserted in the psiCHECK2 vector, which were then co-transfected with miR-552-3p mimics into HEK293 cells. After 24 h of transfection, the HEK293 cells were lysed and subjected to luciferase activity measurement with the Dual Luciferase Assay Kit (Promega, USA) according to the manufacturer's instructions. The firefly and Renilla luciferase activities were determined by a microplate reader. The constitutively expressed firefly luciferase in the vector provided a normalization reference.

### Animal experiments

Male C57BL/6 mice (8‐week age) were purchased from Huafukang Biotechnology Co. Ltd (Beijing, China) and housed in a barrier facility on 12-hour light/12-hour dark cycle with free access to water and normal chow diet.

For diet-induced NASH animal model, the mice fed with a high fat/high-fructose/high-cholesterol diet (HFHFrHC) (Research Diets, Inc. D09100310) for 7 weeks were administered AAV8-Control (n=10) or AAV8-miR-552-3p (n=10) by tail vein injection. After that, mice were kept on HFHFrHC diet and sacrificed after 18 weeks. The blood was collected and livers were harvested for gene expression and histological analysis.

For CCl_4_-induced liver fibrosis animal model, the mice received 5 ml/kg body weight of CCl_4_ (10%) dissolved in olive oil by i.p. injection, three times a week (n=10). To examine the effect of miR-552-3p *in vivo*, 8-week-old mice were administered AAV8-Control (n=10) or AAV8-miR-552-3p (n=10) by tail vein injection. After 8 weeks, they were injected with CCl_4_ in parallel for 2 weeks. All mice were sacrificed to obtain serum and liver samples at 24 h post the last injection of CCl_4_. Furthermore, AAV6-Control and AAV6-miR-552-3p were also used in this model, whose steps were consistent with AAV8.

### Statement on institutional approval for mice experimentation

Animal care, virus injection, and surgical procedures were conducted in compliance with an approved IACUC protocol by Shanghai Institute of Materia Medica, and those set forth in the “Guide for the Care and Use of Laboratory Animals” as published by the National Research Council.

### Hematoxylin-Eosin staining

Hematoxylin-eosin (HE) staining was performed to examine liver tissue morphology according to standard protocols. In brief, liver tissues were fixed with 4% paraformaldehyde overnight and embedded in paraffin. Then, they were sectioned into slices (5 μm) and dehydrated with different concentrations of xylol and ethanol. The cell nuclei were stained with hematoxylin solution for 30 s and the samples were counterstained with eosin solution for 2 min and visualized using optical microscope.

### Detection of ALT, AST, LDL-C

Serum low-density lipoprotein cholesterol (LDL-C), alanine aminotransferase (ALT), and aspartate aminotransferase (AST) were determined by a fully automated biochemical analyzer.

### Hepatic hydroxyproline (HYP) content test

Hydroxyproline (HYP) is one of the main components of collagen and serves as a marker of collagen accumulation in the liver^15^. The content of HYP in livers of model mice were measured by Hydroxyproline Detection Kit (Solarbio, Beijing).

### Masson's trichrome staining, Sirius red staining

Liver tissues were fixed in 4% paraformaldehyde and the 5 μm sections were subjected to Masson's trichrome staining and Sirius red staining to evaluate liver fibrosis. Masson's trichrome staining was performed using standard procedures according to the previous study. Sirius red staining was carried out using an established methodology. Briefly, sections of liver tissue were stained with Sirius red solution for 40 minutes and then washed rapidly with hydrochloric acid (0.5%), and images were obtained using optical microscope.

### Steady-state model insulin resistance index (HOMA-IR) assay

HOMA-IR is an indicator used to evaluate the level of insulin resistance in individuals, calculated as fasting blood glucose level (FPG, mmol/L) × fasting insulin level (FINS, μU/mL)/22.5. Fasting blood glucose testing is performed on mice after 6 h of fasting with a blood glucose monitor when they were fed with HFHFrHC for 20 weeks. Serum insulin levels are measured using the mouse insulin enzyme-linked immune response kit (Jianglai biological, China).

### Immunohistochemistry (IHC) for α-SMA, F4/80 and Ly6G

Immunohistochemical analysis for *α-SMA, F4/80 and Ly6G* were performed using 5 μm‐thick paraffin sections to detect fibrotic area, macrophages and neutrophil respectively. Immunohistochemical procedures were performed using standard procedures according to the previous study^16^.

### Statistical analysis

Statistical analysis was performed with GraphPad Prism 7.0 (GraphPad Software Inc, La Jolla, CA, USA). Statistical significance between 2 groups was assessed by a 2-tailed Student t test. ANOVA was used to compare statistical difference among multiple groups. All the experiments were repeated at least 3 times on separate occasions. p<0.05 was considered to be statistically significant.

## Results

### MiR-552-3p is inversely associated with liver fibrosis or inflammation and downregulated in activated HSCs

**Figure 1:**
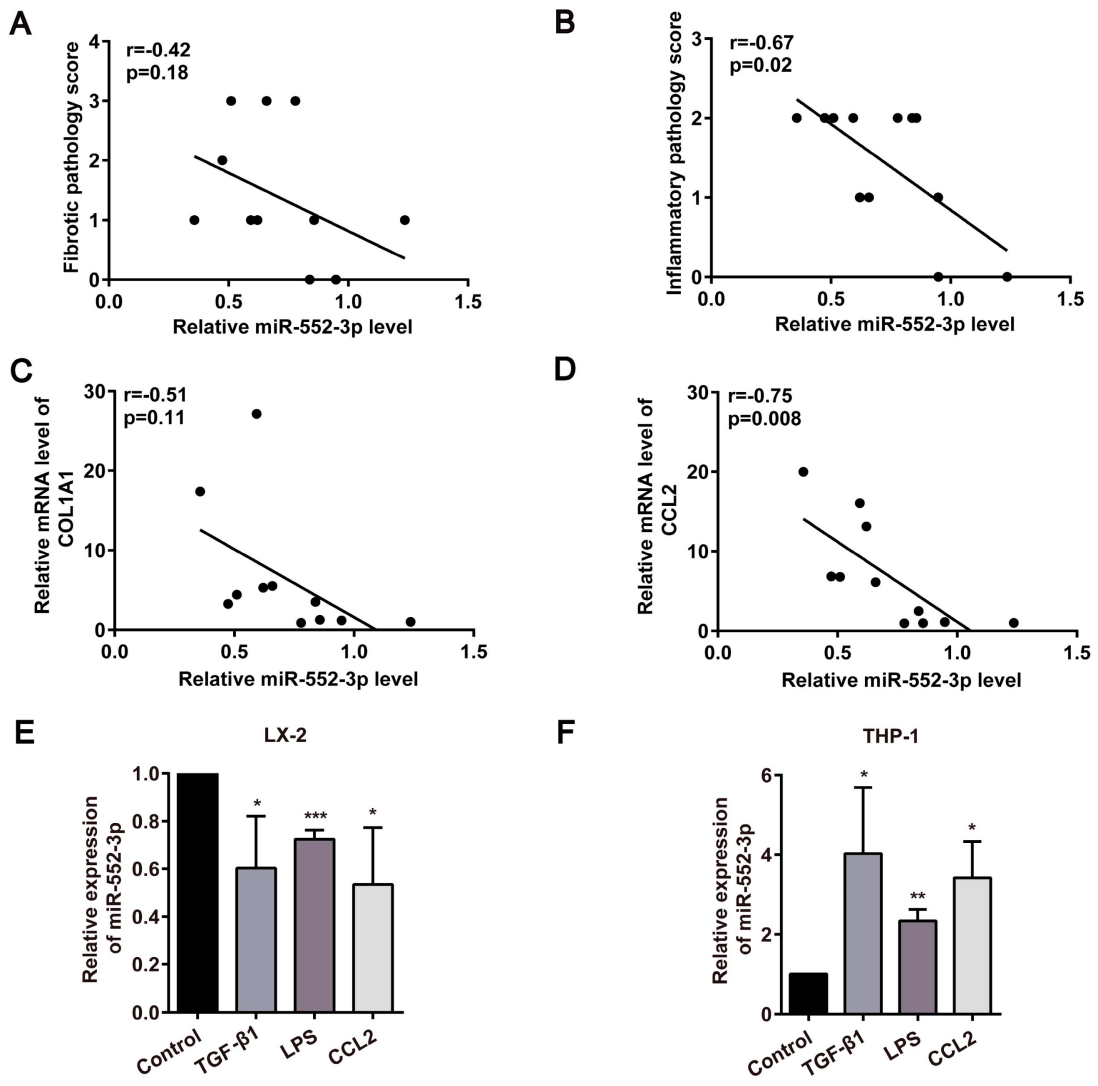
** Negative correlation of miR-552-3p and liver fibrosis or inflammation were discovered in clinical and cell models.** (A-B) Correlation between miR-552-3p content and the pathology scores of fibrosis and inflammation in the liver of patients with NASH (n=12). (C-D) Correlation between miR-552-3p content and the mRNA levels of COL1A1 and CCL2 in the liver of patients with NASH (n=11). (E-F) Expression level of miR-552-3p in LX-2 and THP-1 cells treated with TGF-β1 (10 ng/mL) and CCL2 (50 ng/ mL) for 24 h, LPS (800 ng/mL) for 8h. The miRNA expression was normalized to U6 expression. Data are presented as the mean ± SD of three independent experiments. * P<0.05, ** P<0.01, ***P<0.001 *vs.* Control.

In light of our earlier discovery that miR-552-3p is a negative regulator of hepatic lipid accumulation^14^, we further focused on the function of miR-552-3p on the other pathological features of NASH, especially fibrosis and inflammation. At first, the relationship between miR-552-3p and liver fibrotic and inflammatory pathology scores in NASH patients was analyzed. As shown in Figure 1A-B, the level of hepatic miR-552-3p in the patients with NASH was inversely correlated with the pathologic grading of liver fibrosis or inflammation. Then, by detecting the fibrotic and inflammatory genes in these samples, we discovered that the mRNA levels of *COL1A1* and *CCL2* were also negatively related to the contents of miR-552-3p (Figure 1C-D). Besides, we also analyzed the correlation of miR-552 with fibrotic and inflammatory genes of 39 MAFLD patients and 18 healthy controls from GSE89632 in the Gene Expression Omnibus (GEO) database^17^, where we discovered that miR-552 is inversely correlated with *COL1A1, COL4A2, CCL1* and* TNF* (Figure S1A-D). These results clued the possibility of miR-552-3p regulating liver fibrotic and inflammatory responses of NASH.

HSCs are the main contributors to liver fibrosis by producing most of the ECM when activated, while proinflammatory macrophage activation is highly associated with hepatic inflammation^18^. Moreover, their interplay will continue to remodel immune microenvironment and aggravate the deposition of ECM in the liver. At present, many stimuli have been found to activate HSCs or macrophages, including a series of paracrine/autocrine-mediated cytokines or chemokines (TGF-β1, CCL2, etc.) and gut microflora-derived LPS^19^. We discovered that miR-552-3p was down-regulated significantly in human hepatic stellate cell line (LX-2), while was up-regulated in human leukemia monocytic cell line (THP-1) in response to TGF-β1, CCL2 and LPS stimulation (Figure 1E-F). The consistency between the decrease of miR-552-3p in activated HSCs and its negative correlation with clinical data suggested that miR-552-3p may regulate NASH-associated fibrosis and inflammation via targeting HSCs.

### MiR-552-3p inactivates LX-2 cell through suppressing TGF-β1/Smad3 signaling pathway and attenuates inflammation response induced by LPS

**Figure 2:**
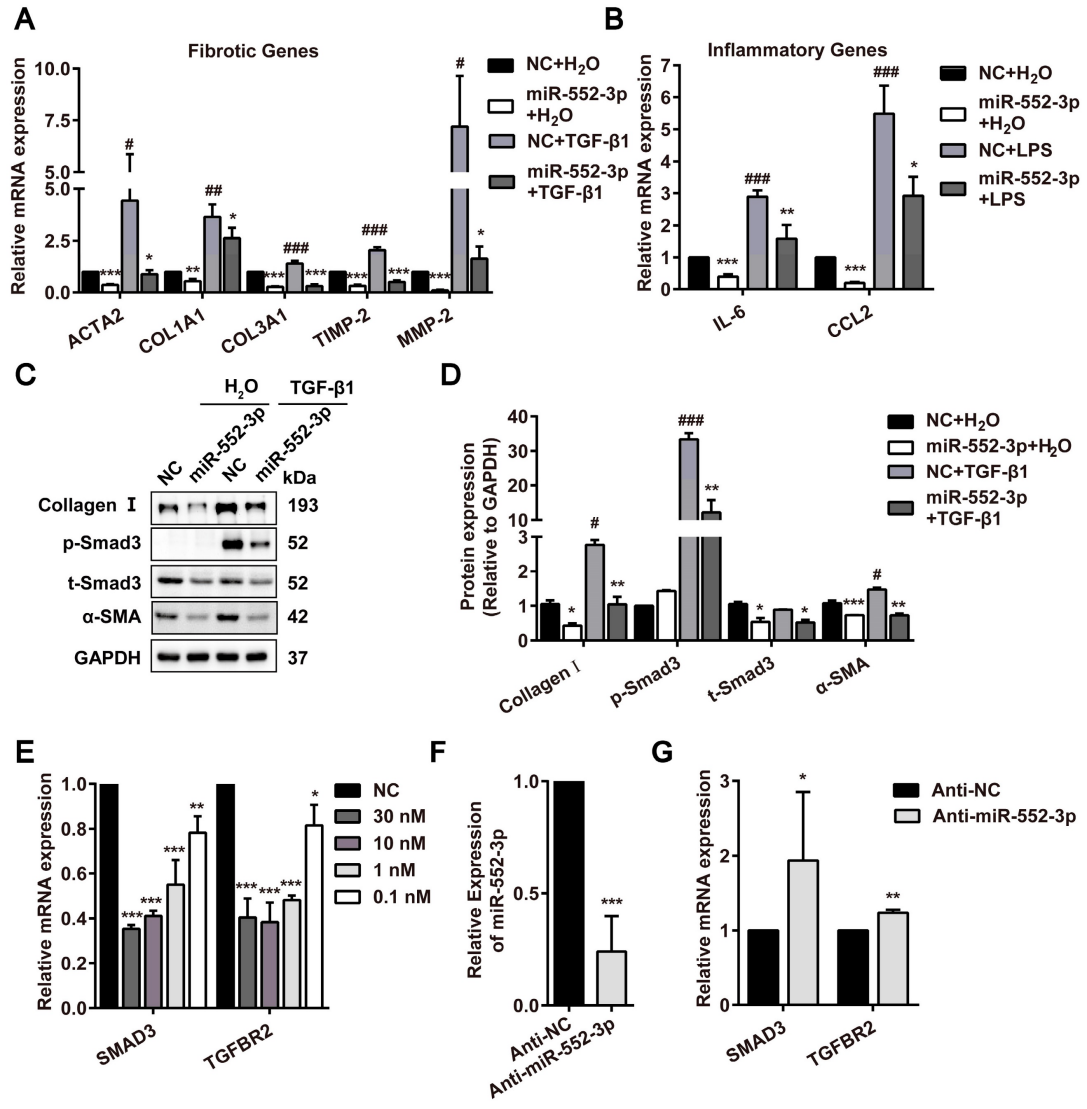
** miR-552-3p regulates TGF-β1/Smad3 signaling pathway in LX-2 cells.** (A) Relative mRNA levels of fibrotic genes in miR-552-3p (1 nM) or NC (1 nM)-transfected LX-2 cells treated with 10 ng/mL TGF-β1 or not for 24 h. (B) Relative mRNA levels of inflammatory genes in miR-552-3p (1 nM) or NC (1 nM)-transfected LX-2 cells treated with 800 ng/mL LPS or not for 8 h. (C-D) The protein levels of Collagen Ⅰ, p-Smad3, t-Smad3 and α-SMA in LX-2 cells transfected with miR-552-3p (1 nM) for 48 h and then treated with 10 ng/mL TGF-β1 or not for 24 h. (E) Relative mRNA levels of SMAD3 and TGFBR2 after the LX-2 cells were transfected with different concentrations of miR-552-3p (30, 10, 1, 0.1 nM) for 48 h. (F) MiR-552-3p level in LX-2 cells transfected with Anti-miR-552-3p or Anti-NC (50 nM) for 72 h. (G) Relative mRNA levels of SMAD3 and TGFBR2 after the LX-2 cells were transfected with Anti-miR-552-3p for 72 h. Data are presented as the mean ± SD of three independent experiments. *P<0.05, ** P<0.01, ***P<0.001 *vs.* NC or Anti-NC; #P<0.05, ##P <0.01, ###P <0.001 *vs.* H_2_O.

As reported, TGF-β1 is a central profibrotic growth factor to promote HSCs activation and liver fibrosis^20^. Additionally, the activation of Τoll-like receptor 4 (TLR4) signaling pathway induced by LPS in HSCs as the molecular link between liver inflammation and fibrosis also has been identified as a key event in liver fibrosis^21^. Thus, we firstly transfected miR-552-3p mimics in LX-2 cells following treated with TGF-β1 or LPS. The results in Figure S2A showed that miR-552-3p can maintain high expression even after 72h transfection. Then, we detected the mRNA levels of fibrotic genes (*ACTA2, COL1A1*, *COL3A1*, *TIMP-2*, *MMP-2*) and inflammatory genes (*IL-6, CCL2*) under the stimulation of TGF-β1 and LPS in LX-2 cells by qPCR, respectively. As expected, the results in Figure 2A-B indicated that miR-552-3p reduced TGF-β1 and LPS-mediated up-regulation of fibrotic and inflammatory genes availably. Furthermore, the LPS of 800ng/ml had no effect on cell livability (Figure S2B).

The aberrant activated TGF-β1/Smad3 signaling pathway has been reported to trigger the activation of HSCs^22^. Therefore, we examined the effects of miR-552-3p on this pathway by western blotting, where miR-552-3p was discovered to attenuate TGF-β1-induced SMAD3 phosphorylation and upregulation of α-SMA and Collagen Ⅰ after treated with TGF-β1 for 24h (Figure 2C). Especially, the effect of miR-552-3p on SMAD3 phosphorylation had already occurred after 15 minutes of TGFβ1 stimulation (Figure S2C). At the same time, we also found that miR-552-3p could lower the total protein level of SMAD3 in LX-2 cells regardless of TGF-β1 stimulation (Figure 2C-D). In the end, we detected the other genes involved in this signaling pathway including SMAD3 by RT-qPCR. The results showed that miR-552-3p could reduce the mRNA levels of intracellular TGFBR2 and SMAD3 at a concentration-dependent manner (Figure 2E), while the TGF-β1, TGFBR1, SMAD2 and SMAD4 were not affected (Figure S3A). To verify the regulatory function of endogenous miR-552-3p on TGFBR2 and SMAD3, we transfected miR-552-3p inhibitor (Anti-miR-552-3p) into LX-2 cells to reduce the level of endogenous miR-552-3p and found the mRNA levels of TGFBR2 and SMAD3 were increased accordingly (Figure 2F-G), which indicated that miR-552-3p suppressed TGF-β1/Smad3 signaling pathway by regulating TGFBR2 and SMAD3. In light of the fact that TLR4 is a pivotal transmembrane protein receptor in LPS-mediated inflammatory signaling, we also measured the mRNA level of TLR4 in LX-2 cells transfected with miR-552-3p, while no change was observed (Figure S3B).

In a word, these results suggested that miR-552-3p negatively mediated LX-2 cell activation through suppressing TGF-β1/Smad3 signaling pathway and attenuated inflammation response induced by LPS without affecting LPS/TLR4 signaling pathway.

### Both endogenous and exogenous miR-552-3p regulate multiple fibrotic and inflammatory genes in LX-2 cells and suppress its proliferation

**Figure 3:**
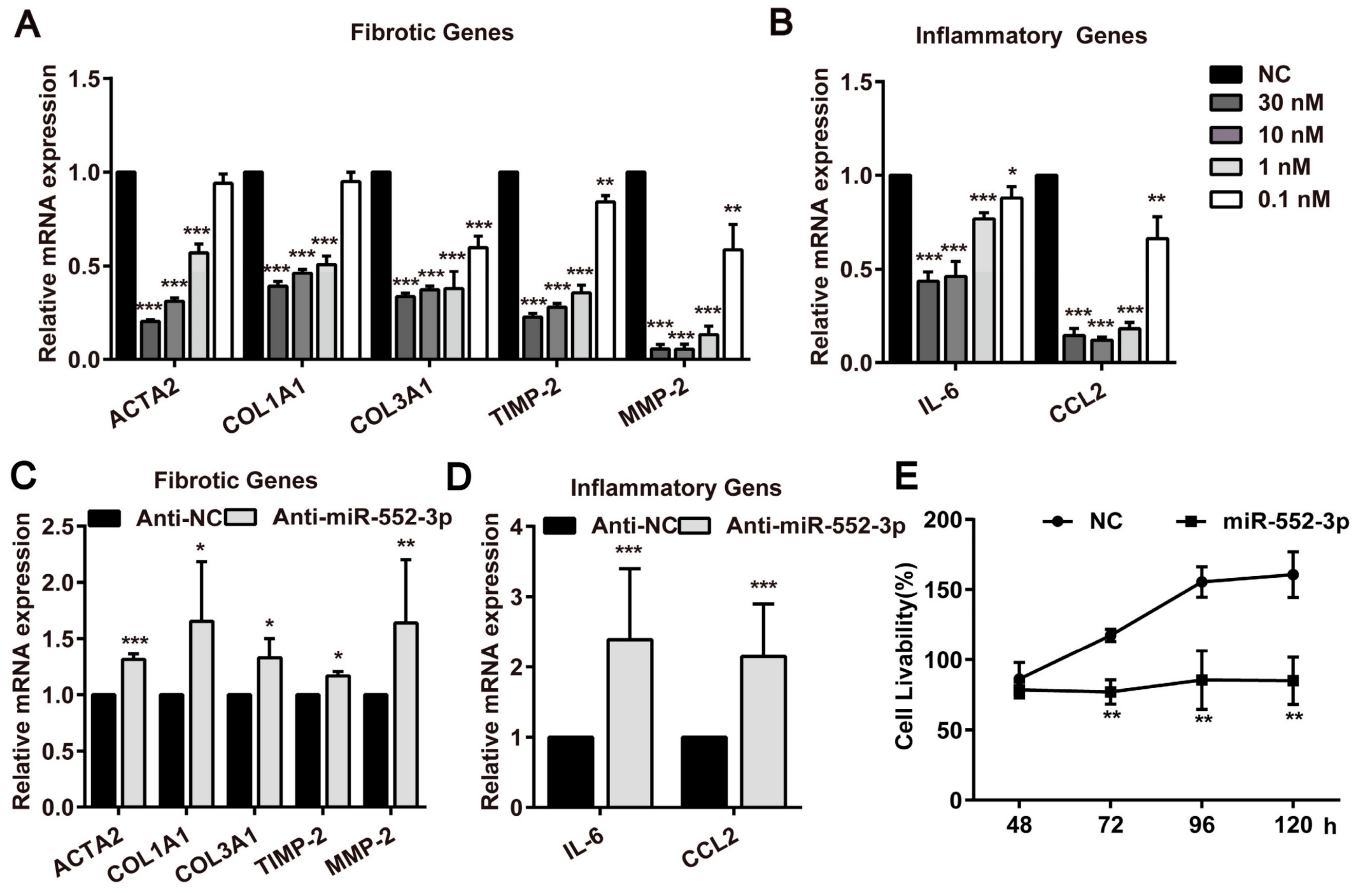
** MiR-552-3p inhibits the expression of fibrotic and inflammatory genes as well as suppresses the proliferation of LX-2 cells.** (A-B) Relative mRNA levels of fibrotic and inflammatory genes in LX-2 cells transfected with different concentrations of miR-552-3p mimics or NC (30, 10, 1, 0.1 nM) for 48 h. (C-D) Relative mRNA levels of fibrotic and inflammatory genes in LX-2 cells transfected with Anti-miR-552-3p (50 nM) for 72 h. The mRNA expression was normalized to GAPDH expression. (E) The cell livability of LX-2 cells transfected with miR-552-3p (1 nM) for 48, 72, 96 and 120 h. Data are presented as the mean ± SD of three independent experiments. *P<0.05, ** P<0.01, ***P<0.001 *vs.* NC or Anti-NC.

During investigating the effect of miR-552-3p on HSCs activated by TGF-β1 and LPS, we noticed that miR-552-3p could also reduce the mRNA of fibrotic and inflammatory genes without the stimuli, suggesting that it may directly target those genes in LX-2 cells. Therefore, we transfected miR-552-3p mimics or inhibitor into LX-2 cells and measured the mRNA levels of these genes. As illustrated in Figure 3A-B, we found the exogenously overexpressed miR‐552-3p resulted in a pronounced down‐regulation of fibrotic and inflammatory genes at concentration-dependent manner, whereas inhibiting intracellular miR-552-3p could elevate mRNA levels of these genes accordingly (Figure 3C-D). Besides, we also discovered that miR-552-3p can suppress the proliferation of HSCs (Figure 3E), which further demonstrated that it can inhibit the activation of LX-2 cells.

Hence, those results proved that miR-552-3p inhibited the activation of LX-2 cells via not only regulating multiple fibrotic and inflammatory genes but also suppressing its proliferation.

### MiR-552-3p relieves liver fibrosis and inflammation in HFHFrHC diet-induced mouse model of NASH

**Figure 4:**
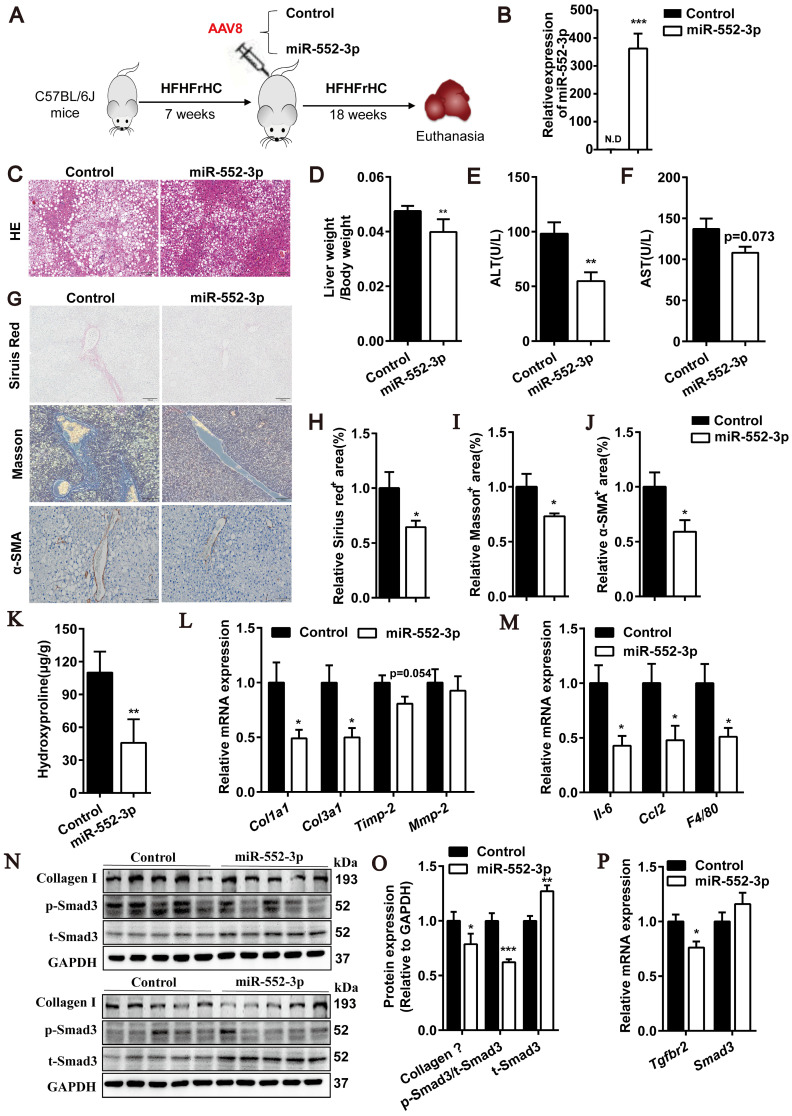
** miR-552-3p down-regulates fibrotic and inflammatory responses in HFHFrHC diet-induced NASH mouse model.** (A) Schematic diagram of HFHFrHC-induced animal experiment. (B) Relative level of miR-552-3p in the liver tissues. (C) HE staining of liver samples. Scale bar: 100 µm. (D) Liver coefficient presented by liver weight to body weight ratio. (E-F) Serum ALT and AST levels. (G) Sirius red, Masson's trichrome and α-SMA staining of liver samples. Scale bar: 100 µm. (H-J) Fibrotic area per field was quantified. (K) Content of hydroxyproline in mouse livers. (L-M) Relative mRNA levels of fibrotic and inflammatory genes in mouse livers. (N-O) Protein levels of Collagen Ⅰ, p-Smad3 and t-Smad3 in TGF-β1/Smad3 signaling pathway. (P) Relative mRNA levels of Tgfbr2, Smad3 in mouse livers. Data are presented as the mean ± SEM. n=10, *P<0.05, ** P<0.01, ***P<0.001* vs.* Control.

After confirmed the inhibitory effect of miR-552-3p on HSCs activation, we were curious of its *in vivo* function in regulating liver fibrosis and inflammation. Firstly, an animal model of NASH induced by HFHFrHC diet was used to study the gain-of-function of miR-552-3p, where the mice pre-fed with HFHFrHC diet for 7 weeks were injected with AAV8-miR-552-3p and fed with this diet for another 18 weeks (Figure 4A). As shown in Figure 4B, AAV8 could successfully overexpress miR-552-3p in the livers of mice. Consistent with the reported results^14^, miR-552-3p can alleviate hepatic glycolipid metabolism disorder featured by reduced lipid droplet deposition in HE staining (Figure 4C), lower LDL-C and fasting blood glucose in blood, and improved HOMA-IR index (Figure S4A-C). More importantly, miR-552-3p could also decrease liver coefficient (liver weight/body weight) without affecting the body weight (Figure 4D and Figure S4D), and suppress the increase of ALT and AST (Figure 4E-F), which suggested that it can alleviate liver injury stimulated by HFHFrHC diet. Notably, the serum ALT which has been proved to predict the presence of liver fibrosis and be a risk for fibrosis progression in NASH^23^ was reduced markedly (Figure 4E).

Since some studies have shown that HFHFrHC diet can induce liver inflammation and fibrosis in mice after 25 weeks of induction^24^, we further explored the effects of miR-552-3p on liver fibrosis and inflammation. Firstly, the Sirius red and Masson's trichrome staining were performed for pathological evaluation of fibrosis. It was clearly observed that collagen deposition which was the marker of liver fibrosis was improved by supplementing with miR-552-3p (Figure 4G-I). Moreover, α-SMA levels in liver tissues, another marker of liver fibrosis, were detected by immunohistochemical staining. Consistently, lower α-SMA expression was also appeared in mouse livers with miR-552-3p overexpression (Figure 4G and J). Then, as shown in Figure 4K, hepatic hydroxyproline content was decreased significantly in miR-552-3p group, which confirmed that miR-552-3p can inhibit the formation of collagen fiber. The qPCR results showed that overexpression of miR-552-3p significantly reduced the mRNA levels of fibrotic genes (*Col1a1*, *Col3a1*, *Timp-2*) and inflammatory genes (*Il-6*, *Ccl2*, *F4/80*) in mice livers, which demonstrated that miR-552-3p could reduce liver fibrosis and inflammation in mice again (Figure 4L-M). In order to further clarify the effect of miR-552-3p on liver inflammation, we determined the histological NAS score of inflammatory and the result in Figure S4E showed that it could decrease the inflammatory score in this NASH model. In addition, we detected the population of neutrophils and macrophages in the liver through immunohistochemistry with anti-F4/80 and anti-Ly6G. The results suggested that miR-552-3p could reduce the number of macrophages without influencing neutrophil (Figure S4F). Finally, we detected the effect of miR-552-3p on the TGF-β1/Smad3 signaling pathway *in vivo*. The Western blotting results showed that miR-552-3p could significantly reduce Collagen Ⅰ and the ratio of p-Smad3 versus t-Smad3 (Figure 4N-O), while RT-qPCR results showed the mRNA level of Tgfbr2 in the liver was also obviously decreased, which was consistent with the *in vitro* results (Figure 4P). However, unlike cell experiments, miR-552-3p can increase protein level of Smad3 and had no effect on the mRNA level of Smad3, which might be due to the differences between human and mouse or the insufficient content of miR-552-3p delivered to hepatic stellate cells by AAV8.

Overall, these results demonstrated that miR-552-3p can relieve the liver fibrotic and inflammatory responses of NASH mouse model induced by HFHFrHC diet and participate in the regulation of the TGF-β1/Smad3 signaling pathway *in vivo*.

### MiR-552-3p alleviates liver fibrosis and inflammation in CCl_4_-induced mouse model via regulating HSCs directly

Next, in order to rule out whether the function of miR-552-3p in relieving liver fibrosis and inflammation is due to its role in improving glycolipid metabolism disorders, we used another classical model of liver fibrosis induced by CCl_4_ to clarify it. Mice were injected with AAV8-miR-552-3p or AAV8-Control via tail vein and allowed an 8-week expression period, followed by intraperitoneal injection of CCl_4_ (5 mL/kg, dissolved into olive oil) three times a week for 2 weeks (Figure S5A). As shown in Figure S5B-F, the overexpressed miR-552-3p in the liver of mice could improve liver fibrosis phenotype induced by CCl_4_. The ALT and AST levels in the serum showed that miR-552-3p could attenuate the liver injury in this model (Figure S5G-H). Meanwhile, we also observed the same phenomena that miR-552-3p significantly suppressed the hydroxyproline content (Figure S5I), reduced the mRNA levels of fibrotic genes (*Col1a1*, *Col3a1*, *Timp-2*, *Mmp-2*) and inflammatory genes (*Il-6*, *CCL2*, *F4/80*) (Figure S5J-K). Finally, by testing the protein levels of Collagen Ⅰ and p-Smad3 with Western blotting and the mRNA levels of Tgfbr2 and Smad3 with RT-qPCR, we clarified that miR-552-3p also participated in regulating the TGF-β1/Smad3 signaling pathway in this mice model (Figure S5L-N). These results indicated that miR-552-3p can directly alleviate the liver fibrotic and inflammatory responses of liver fibrosis animal model induced by CCl_4_, independent of its improvement on lipid accumulation.

**Figure 5:**
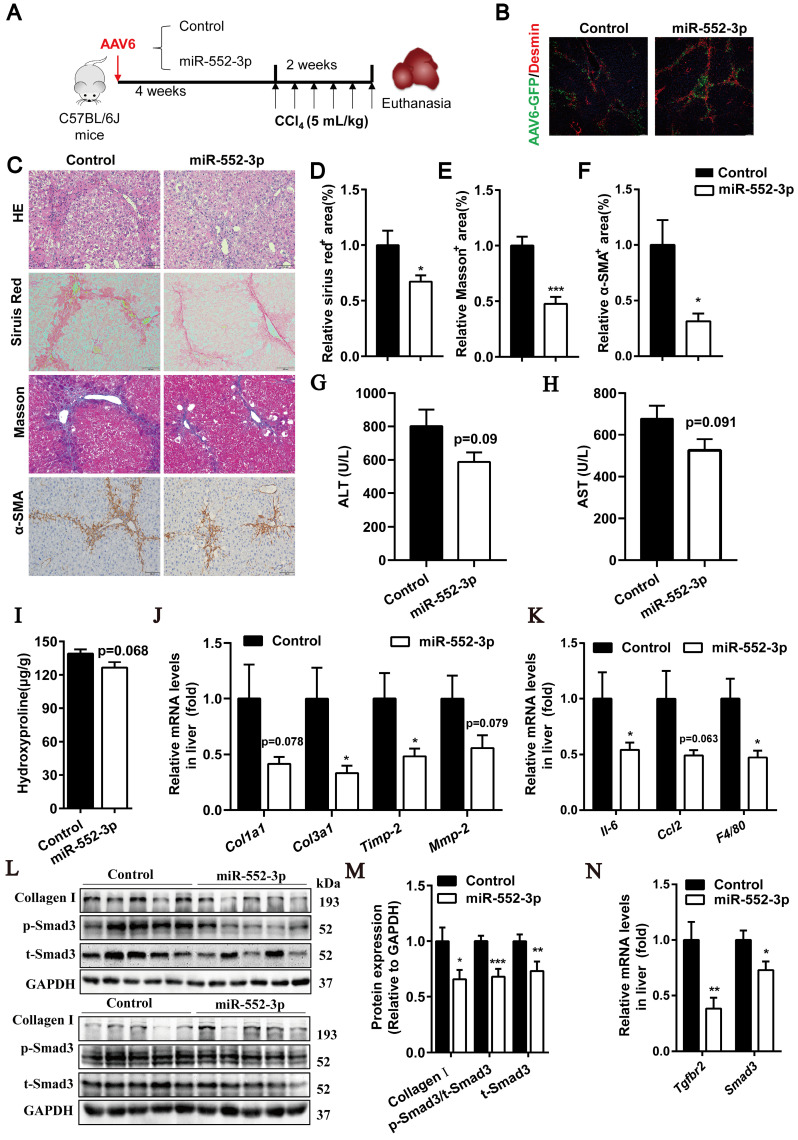
** miR-552-3p overexpressed by AAV6 alleviates liver fibrosis and inflammation in CCl_4_-induced animal model.** (A) Schematic diagram of CCl_4_-induced animal experiment. (B) The Desmin staining of liver tissues. (C) HE, Sirius red, Masson's trichrome and α-SMA staining of liver samples. Scale bar: 100 µm. (D-F) Fibrotic area per field was quantified. (G-H) ALT and AST levels in mouse serum. (I) The content of hydroxyproline in mouse livers. (J-K) Relative mRNA levels of fibrotic and inflammatory genes in mouse livers. (L-M) Protein expression levels of Collagen Ⅰ, p-Smad3 and t-Smad3 in mouse livers. (N) Relative mRNA levels of Tgfbr2 and Smad3 in mouse livers. Data are presented as the mean ± SEM. n=10, *P<0.05, ** P<0.01, ***P<0.001 *vs.* Control.

In addition, to further demonstrate whether miR-552-3p can affect liver fibrosis and inflammation by regulating the activation of hepatic stellate cells *in vivo*, we used AAV6 to overexpress miR-552-3p in hepatic stellate cells specifically^25^, ^26^. Mice were injected with AAV6-miR-552-3p via tail vein and allowed a 4-week expression period, followed by intraperitoneal injection of CCl_4_ (5 mL/kg, dissolved into olive oil) three times a week for 2 weeks (Figure 5A).

As shown in Figure 5B, AAV6 could transfer miR-552-3p in HSCs successfully. The HE, Sirius red, Masson's trichrome and α-SMA staining showed in Figure 5C-F suggested that it could alleviate the extent of liver fibrosis effectively in this mouse model. The decreased levels of ALT and AST further demonstrated that miR-552-3p had the function of relieving liver damage (Figure 5G-H). Meanwhile, we also observed that miR-552-3p significantly suppressed the hydroxyproline content (Figure 5I), reduced the mRNA levels of fibrotic genes (*Col1a1*, *Col3a1*, *Timp-2*, *Mmp-2*) and inflammatory genes (*Il-6*, *Ccl2*, *F4/80*) (Figure 5J-K). Finally, we found that miR-552-3p could remarkably decrease the protein levels of p-Smad3 and Collagen Ⅰ as well as the mRNA levels of Tgfbr2, which is as same as the results of when miR-552-3p delivered by AAV8. In addition, we also found that the mRNA and protein levels of smad3 were downregulated in the miR-552-3p group, which was different from the data in the previous two animal models (Figure 5L-N) and consistent with the *in vitro* results (Figure 2C-E). This result further verified that AAV6 is the most suitable carrier for targeting hepatic stellate cells.

In a word, these results confirmed that miR-555-3p could improve liver fibrosis and inflammation through inactivating HSCs.

### The inhibitory effect of miR-552-3p on fibrotic and inflammatory genes may not occur in the nucleus

**Figure 6:**
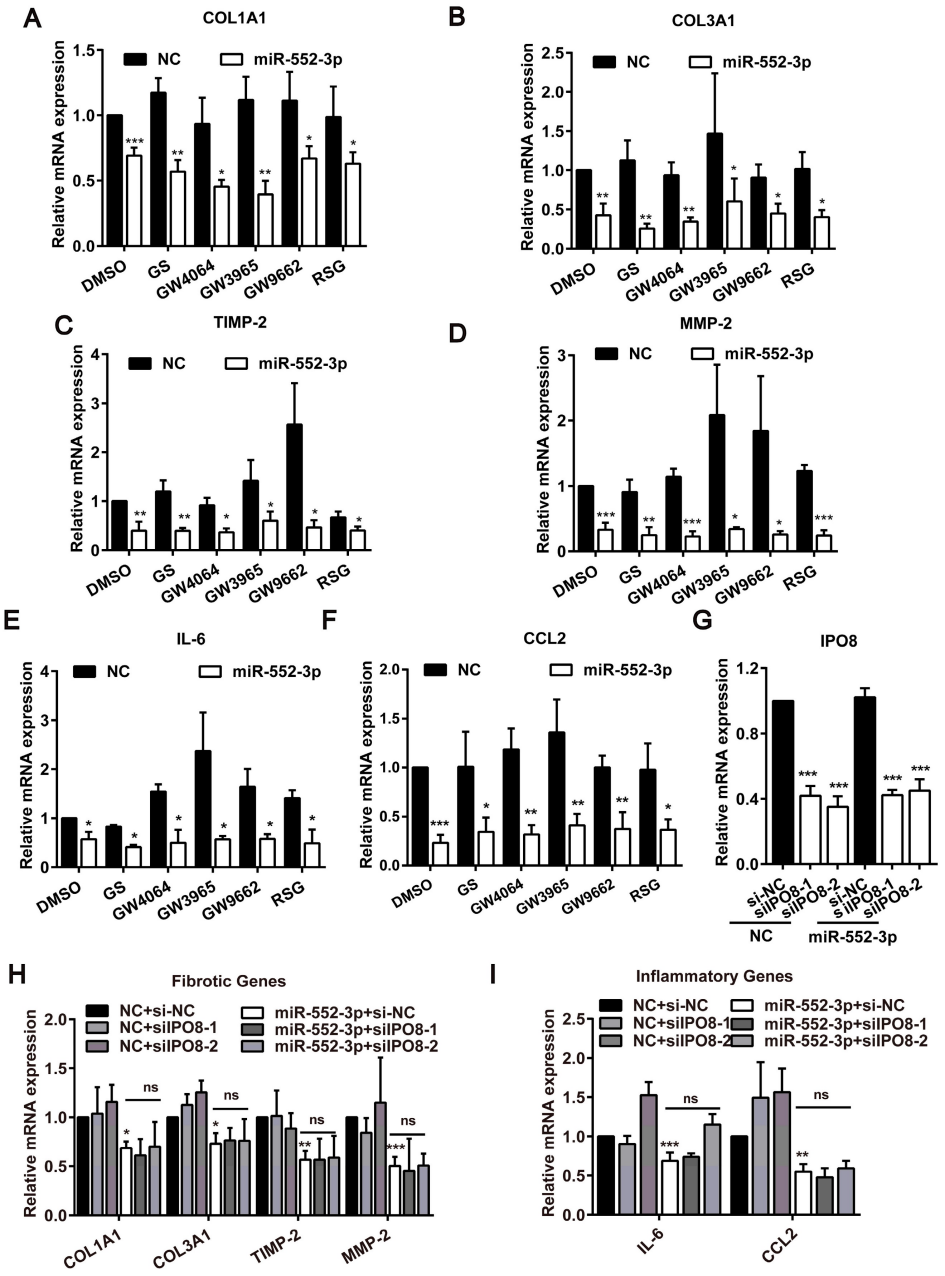
** Function of miR-552-3p in suppressing fibrotic and inflammatory genes may not occur in the nucleus.** (A-F) Relative mRNA levels of fibrotic and inflammatory genes in LX-2 cell transfected with miR-552-3p mimics (1 nM) for 24 h and then treated with agonists or antagonists of FXR, LXRα and PPARγ for 24 h. GS (10 μM): FXR antagonist; GW4064 (2 μM): FXR agonist; GW3965 (2 μM): LXRα agonist; GW9662 (1 μM): PPARγ antagonist; RSG (5 μM): PPARγ agonist. (G) Relative mRNA levels of IPO8 in LX-2 cell transfected with 2 different sequences of siIPO8 (30 nM) for 48 h. (H-I) Relative mRNA levels of fibrotic and inflammatory genes in LX-2 cell co-transfected with siIPO8 (30 nM) and miR-552-3p mimics (1 nM) for 48 h. Data are presented as the mean ± SD of three independent experiments. *P<0.05, ** P<0.01, ***P<0.001 *vs.* NC; ns, non-significant.

The aforementioned findings identified miR-552-3p could attenuate hepatic fibrosis and inflammation *in vivo* and *in vitro*, but the particular mechanism need to be explored further. In the light of the previous report that miR-552-3p regulates the transcriptional activity of nuclear receptor subfamily 1 (NR1) including FXR, LXRα and PPARγ which participate in the liver fibrosis and inflammation processes by promoting the transformation of HSCs^27^, we firstly examined the effect of miR-552-3p on the mRNA levels of fibrotic and inflammatory genes under the condition of agonist or antagonist of FXR, LXRα and PPARγ^14^. Unexpectedly, the inhibitory activity of miR-552-3p on these genes still existed, which suggested that its inhibition of these fibrotic and inflammatory genes did not depend on its role in regulating these nuclear receptors (Figure 6A-F).

Since we have ever discovered that miR-552-3p can be localized in the nucleus to modulate gene transcription^14^, we then reduced IPO8 level (a key gene responsible for miRNA transporting to the nucleus) using siRNA to decrease the level of miR-552-3p in the nucleus and detected whether the anti-fibrotic and anti-inflammatory function of miR-552-3p were occurred in the nucleus (Figure 6C). As illustrated in Figure 6H-I, the inhibitory effect of miR-552-3p on these genes were not affected while we decreased the miR-552-3p level in the nucleus.

### MiR-552-3p inhibits fibrotic and inflammatory genes through its seed sequence binding with 3'-UTR of these genes

**Figure 7:**
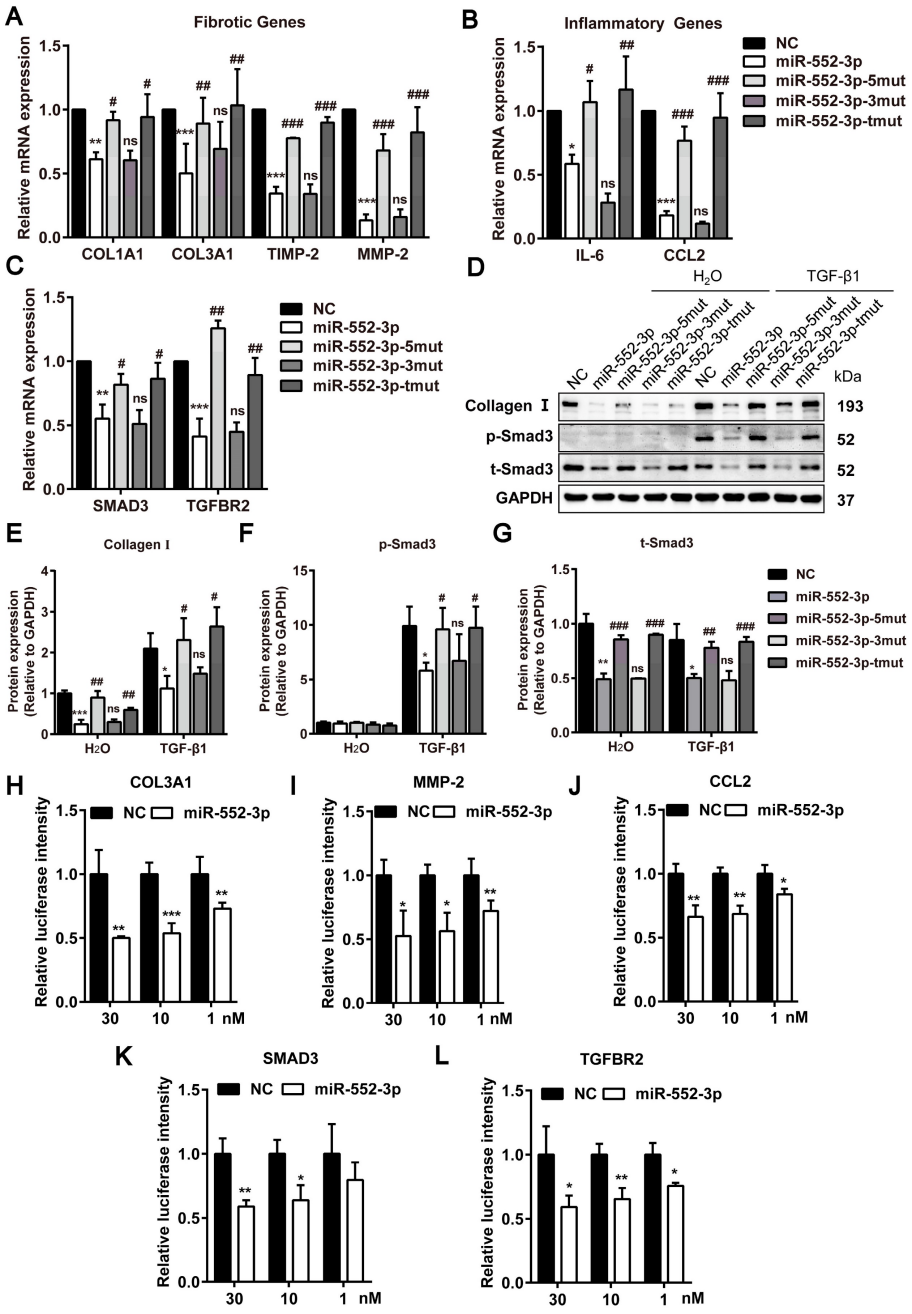
** miR-552-3p inhibits fibrotic and inflammatory genes through the seed sequence binding to their 3'-UTR.** (A-C) Relative mRNA levels of fibrotic and inflammatory genes in LX-2 cells transfected with miR-552-3p and its 3 mutants (1 nM) for 48 h. (D-G) The protein levels of Collagen I, p-Smad3 and t-Smad3 in TGF-β1/Smad3 signaling pathway in LX-2 cells transfected with miR-552-3p and its 3 mutants (1 nM) for 72 h. (H-L) The activity of luciferase in HEK293 cells transfected with 3'-UTR reporter of COL3A1, MMP-2, CCL2, SMAD3 and TGFBR2 (psiCHECK2-COL3A1/MMP-2/CCL2/SMAD3/TGFBR2) and miR-552-3p or the corresponding NC (30, 10, 1 nM). Data are presented as the mean ± SD of three independent experiments. *P<0.05, ** P<0.01, ***P<0.001 *vs*. NC; #P<0.05, ##P <0.01, ###P <0.001 *vs*. miR-552-3p; ns, non-significant.

The seed sequence located in the position 2-8 of the miRNA is conservative and has a major contribution to the function of miRNAs. To figure out the contribution of seed and non-seed sequence of miR-552-3p on its function in inhibiting fibrotic and inflammatory genes, we synthesized the mutants of seed sequence (miR-552-3p-5mut), non-seed sequence (miR-552-3p-3mut) and both regions (miR-552-3p-tmut) respectively. The sequences of three mutants were displayed in Table 1. Then, we transfected these mutants into LX-2 cells and tested the mRNA levels of fibrotic and inflammatory genes by RT-qPCR. As expected, the inhibitory function of miR-552-3p on these genes disappeared in miR-552-3p-5mut and miR-552-3p-tmut groups which contained the mutations in the seed sequence (Figure 7A-B). Similarly, we also found that miR-552-3p could not affect the TGF-β1/Smad3 signaling pathway (Figure 7D-G, S6A) and LX-2 proliferation (Figure S6B) when its seed sequence was mutated.

The post-transcriptional repression through miRNA seed sequence binding to 3'-UTR of target mRNA is considered as the canonical mode of miRNA-mediated gene regulation^28^. Through sequence alignment, we found that there were potential binding sites in these genes with the seed sequence of miR-552-3p (Table 2). To further explore whether the miR-552-3p directly targets to the mRNAs of these genes, the luciferase reporter assays were performed. We conducted the dual luciferase experiments to verify this hypothesis using COL3A1, MMP-2, CCL2, SMAD3 and TGFBR2 as examples. As shown in Figure 7H-L, we found that miR-552-3p could inhibit the luciferase activities of the 3'-UTR of three genes at a concentration-dependent manner.

In conclusion, these results suggested that miR-552-3p could inhibit fibrotic and inflammatory genes through seed sequences by binding to their 3'-UTR.

## Discussion

**Figure 8:**
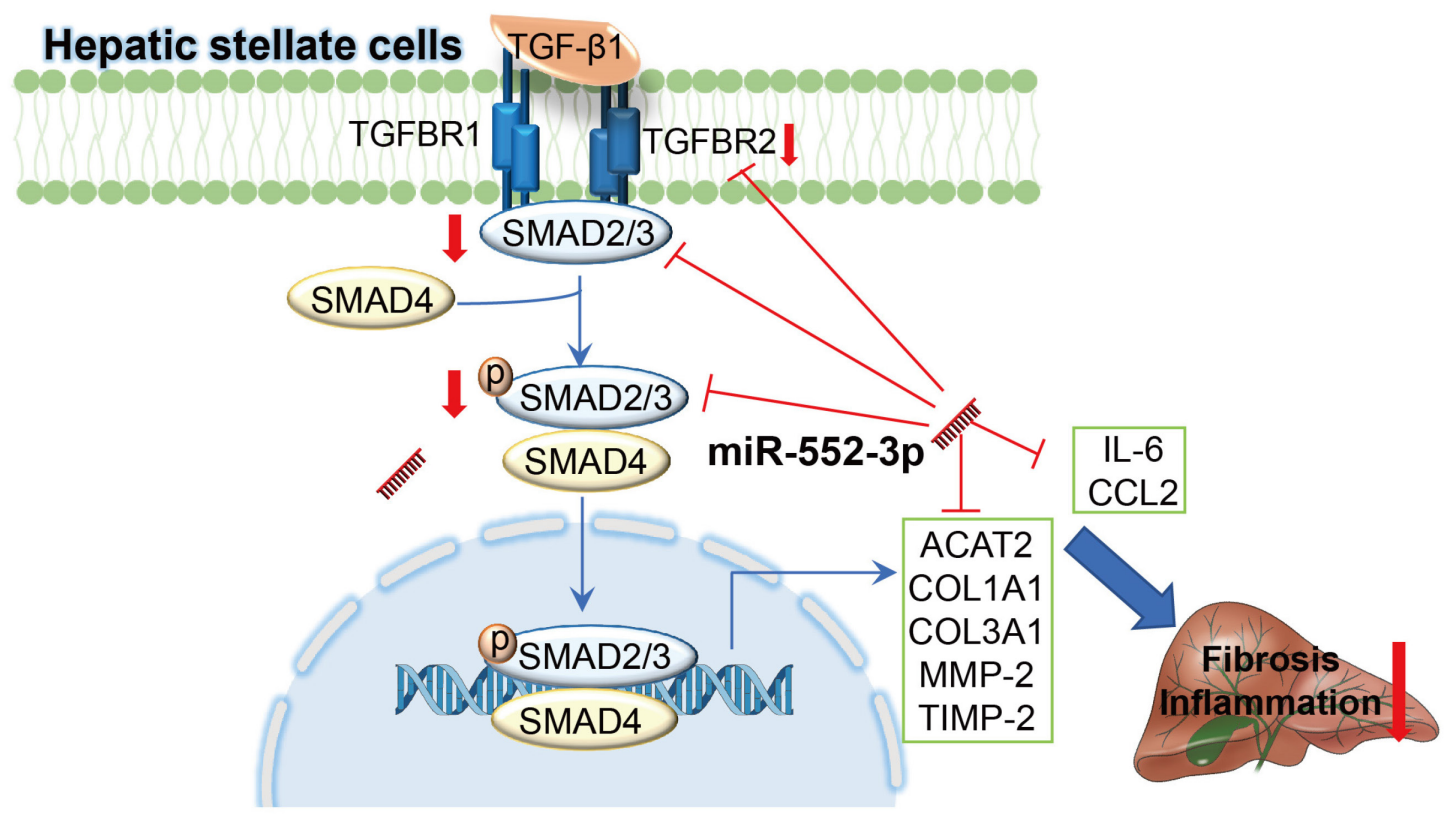
** Schematic representation of the mechanism by which miR-552-3p alleviates liver fibrosis and inflammation.** MiR-552-3p alleviates liver fibrosis and inflammation via inactivating hepatic stellate cells. miR-552-3p targets the 3'-UTRs of TGFBR2 and SMAD3 to inhibit TGF-β1/Smad3 pathway and decrease the expression of fibrotic genes indirectly, and targets to the 3'-UTRs of multiple fibrotic and inflammatory genes to decrease their expression directly, thus suppressing HSC activation and proliferation.

As the more end-stage of MAFLD, NASH phenotype is characterized by hepatic steatosis, inflammation and fibrosis. It is a key stage from hepatosteatosis to fibrosis/cirrhosis and eventually HCC^29^, whose underlying pathogenesis attracted increasing considerable attentions. We reported here for the first time that the hepatic miR-552-3p level of NASH patients is negatively correlated to the liver fibrosis and inflammation degrees. Firstly, by overexpressing exogenous miR-552-3p with mimics and inhibiting endogenous miR-552-3p with inhibitor in LX-2 cells, we confirmed that miR-552-3p could affect the activation of HSCs via regulating the expression of multiple fibrotic genes (*ACTA2*, *COL1A1*,* COL3A1*, *TIMP-2*, *MMP-2*), inflammatory genes (*IL-6*, *CCL2*), and some genes in TGF-β1/Smad3 signaling pathway (*TGFBR2* and *SMAD3*) *in vitro*. Unfortunately, miR-552-3p is only discovered in humans and non-human primates, which makes it impossible to study the *in vivo* role of endogenous miR-552-3p in mice. Since miR-552-3p can bind to the 3'-UTR of fibrotic and inflammatory genes of both human and mouse predictably (Table 2), we investigated its protective function in two mouse models by AAV delivery system: diet-induced animal model of NASH by HFHFrHC and CCl_4_-induced animal model of liver fibrosis (Figure 8). Therefore, we supposed that miR-552-3p might play a crucial role in maintaining the quiescent condition of HSCs and its reduction might be one of the pathologic causes of NASH.

It is widely accepted that inflammation and fibrosis are two main features during NASH development. Fibrosis is a highly conserved response to hepatic injury and HSCs are the major contributors as they promote collagen deposition in ECM once activated^30^. Other than the normal physiological functions on hepatic fibrosis, HSCs also participate in hepatic inflammation by releasing a battery of inflammatory cytokines and chemokines, as well as is highly responsive to pro-inflammatory cytokines and LPS^31^. This suggests that regulating the function of HSCs is of great significance to improve the pathological characteristics of NASH. Recently, some miRNAs have been found to play multifaceted roles in HSC activation, proliferation and production of ECM^32^. In our work, we firstly discovered that miR-552-3p was reduced when LX-2 cells were activated by TGF-β1, CCL2 and LPS. Moreover, the *in vitro* experiments showed that miR-552-3p indeed down-regulated the expression of multiple fibrotic and inflammatory genes. In addition to inhibiting HSCs activation, the overexpression of miR-552-3p significantly inhibited HSCs proliferation, indicating that miR-552-3p plays an important role in regulating the function of HSCs. Using the AAV8 delivery system, we successfully overexpressed miR-552-3p in two animal models and demonstrated its ability to alleviate liver fibrosis and inflammation. However, AAV8 is well known mostly to target hepatocytes in the liver, not HSCs. Thus, the function of AAV8-miR-552-3p may contribute to the delivery of miR-552-3p from the extracellular vesicles secreted by hepatocytes to HSCs. Then, in order to further demonstrate that miR-552-3p exerts this function by directly regulating hepatic stellate cells, we used AAV6 to transfer miR-552-3p in HSCs in CCl_4_ induced animal model. Consistent with our conjecture, the miR-552-3p delivered by AAV6 had a more significant inhibitory effect on liver fibrosis and inflammation. What's more, it could inhibit smad3 expression which was not observed in AAV8 animals. Additionally, the alleviating effect of miR-552-3p on inflammation and fibrosis *in vivo* clued the potential application of regulating HSCs in the treatment of NASH once again.

It has been reported that hepatocytes, a variety of immune cells (including hepatic macrophages and lymphocyte), and their crosstalk with HSCs play a vital role in hepatic fibrosis of NASH. Briefly, steatotic hepatocyte will secrete fibrogenic injury signals to activate HSCs and Kupffer cells, and recruit lymphocytes, then these activated cells further secrete profibrogenic and proinflammatory mediators to aggravate liver fibrosis. That is to say, the hepatocyte-macrophage-HSC network is at the center of the fibrogenic response in NASH^4^, ^33^. Confusingly, we found that the level of miR-552-3p was reduced in LX-2 cells while increased in THP-1 cells, which suggested that it may have different functions in different cell types. Nevertheless, it is necessary to conduct more experiments to prove it because THP-1 cell is a type of monocyte which is not same as the Kupffer cells in the liver. The benefit effect of miR-552-3p on liver fibrosis induced by CCl_4_ suggested that its role of improving glycolipid stress in hepatocytes did not contribute to its inhibition in HSCs activation. However, more evidence needs to be unearthed to elaborate clearly whether the effect of miR-552-3p on relieving liver fibrosis and inflammation is related to its expression in other types of cells.

TGF-β1 is a central regulator of liver fibrosis, and the TGF-β/SMAD signaling pathway is probably the most prominent direct inducer of HSCs activation. Suppression of the activity of TGF-β/SMAD signaling by many therapeutic strategies shows anti-fibrotic effects in fibrotic diseases. We found that miR-552-3p inhibited p-Smad3 level induced by TGF-β1, suggesting that it could lead to the suppression of TGF-β1/Smad3 signaling. Interestingly, miR-552-3p over-expression also resulted in the suppression of TGFBR2 and SMAD3. Accumulating evidence has clarified that knockdown of TGFBR2 or SMAD3 could induce the inactivation of TGF-β1/Smad3 pathway. Our further studies confirmed that TGFBR2 and SMAD3 were targets of miR-552-3p, demonstrating that miR-552-3p inhibits TGF-β/Smad3 signaling, at least in part, via TGFBR2 and SMAD3. Confusingly, the stimuli of THP-1 increased the level of miR-552-3p, which promoted us to hypothesize that the ability of miR-552-3p in relieving inflammation may not due to the effect on macrophages. In addition, we also discovered that miR-552-3p could suppress the TGF-β1/Smad3 signaling pathway, while did not affect the LPS/TLR4 signaling pathway. Therefore, it is necessary to explore whether there are other signaling pathways involved in inflammation influenced by miR-552-3p. Furthermore, how TGF‐β1, CCL2 and LPS reduced the expression of miR‐552-3p and whether other cytokines involved in fibrosis and inflammation influence the content of miR-552-3p also need further investigation.

Finally, through the *in vitro* and *in vivo* experiments, we discovered that miR-552-3p could inactivate HSCs and improve liver fibrosis and inflammation in the mouse models. However, whether miR-552-3p has the potential to treat fibrosis and inflammation still need more evidence, for example, to examine its effect in other liver fibrosis mouse model and a model of rhesus monkeys.

In conclusion, our study found that miR-552-3p could not only relieve glycolipid metabolism which has been confirmed previously, but also ameliorate liver fibrosis and inflammation of NASH. These results provide novel insight that miR-552-3p might be involved in the pathogenesis of NASH by regulating hepatic stellate cells and the therapeutic potential of miR-552-3p in NASH treatment.

## Supplementary Material

Supplementary figures and tables.

## Figures and Tables

**Table 1:** Sequences of miR-552-3p and its mutants

miR-552-3p	AACAGGUGACUGGUUAGACAA
miR-552-3p-5mut	AAC**GAAC**G**U**CUGGUUAGACAA
miR-552-3p-3mut	AACAGGUGACU**AAC**U**C**GACAA
miR-552-3p-tmut	AAC**GAAC**G**U**CU**AAC**U**C**GACAA

**Table 2:** Sequence alignment of miR-552-3p and the 3'-UTR of ACTA2, COL1A1, COL3A1, MMP-2, TIMP-2, SMAD3, TGFBR2, IL-6 and CCL2

Target gene	AACAGAUUGGUC*AGUGGACAA*
**ACTA2**	CAACTGTGAATGT**CCTGT**GGAATTATGCCT
miR-552-3p	AACAGAUUGGUCAGU***GGACA***A
**COL1A1**	cccucucuc**caccug**ccucuggcuuc
miR-552-3p	AACAGAUUGGUCA***GUGGAC***AA
**COL3A1**	uccuucau**ccugu**aaaggucaac
miR-552-3p	AACAGAUUGGUCAGU***GGACA***A
**COL3A1**	augacugugc**ucacc**aguaaaagaua
miR-552-3p	AACAGAUUGGUC***AGUGG***ACAA
**MMP-2**	acuuuucacgu**gucacc**uauuuuaacc
miR-552-3p	AACAGAUUGGU***CAGUGGA***CAA
**TIMP-2**	UCUUGUUUCCUCC**CACCUGU**G
	GGCAACCCCAAAG**CACCUGU**U
miR-552-3p	AACAGAUUGGUCA***GUGGACA***A
**SMAD3**	GAAGGGGUGCACC**CACCUGU**U
	UUGUAGGCAAACC**CACCUGU**G
	CUUAAAAGCAGAG**CACCUGU**U
miR-552-3p	AACAGAUUGGUCA***GUGGACA***A
**SMAD3**	aaguggcgu**ucaccu**agucaaca
	gccuucac**ucaccu**ucacugcug
	ugacaucu**ucaccu**ugcagcu
miR-552-3p	AACAGAUUGGUC***AGUGGA***CAA
**TGFBR2**	CCUAACUUAAUUA**CACCUGU**A
miR-552-3p	AACAGAUUGGUCA***GUGGACA***A
**IL-6**	ucuggucagaa**accugu**ccacugggca
miR-552-3p	AACAGAUUGGUCAG***UGGACA***A
**CCL2**	cccagacac**ccuguu**uuauuuuau
miR-552-3p	AACAGAUUGGUCAGU***GGACAA***

## Abbreviations

NAFLD: Non-alcoholic fatty liver disease
MAFLD: Metabolic-associated fatty liver disease
NASH: Non-alcoholic steatohepatitis
HCC: Hepatocellular carcinoma
HSCs: Hepatic Stellate Cells
ECM: Extracellular Matrix
TGF-β1: Transforming growth factor β1
miRNAs: MicroRNAs
CCl4: Carbon tetrachloride
PMA: Phorbol 12-myristate 13-acetate
HE: Haematoxylin-eosin
LDL: Low-density Lipoprotein Cholesterol
ALT: Alanine Aminotransferase
AST: Aspartate Aminotransferase
HYP: Hydroxyproline
IHC: Immunohistochemistry
GEO: Gene Expression Omnibus
TLR4: Τoll-like Receptor 4
NR1: Nuclear Receptor Subfamily 1
