# Supplementary material for: MiR-552-3p Regulates Multiple Fibrotic and Inflammatory genes Concurrently in Hepatic Stellate Cells Improving NASH-associated Phenotypes
Source: Int J Biol Sci. 2023 Jul 3;19(11):3456–71. doi: 10.7150/ijbs.80760 (PMC10367551; doi:10.7150/ijbs.80760)
Supplement: Supplementary file 1 — Supplementary figures and tables. [file ijbsv19p3456s1.pdf]

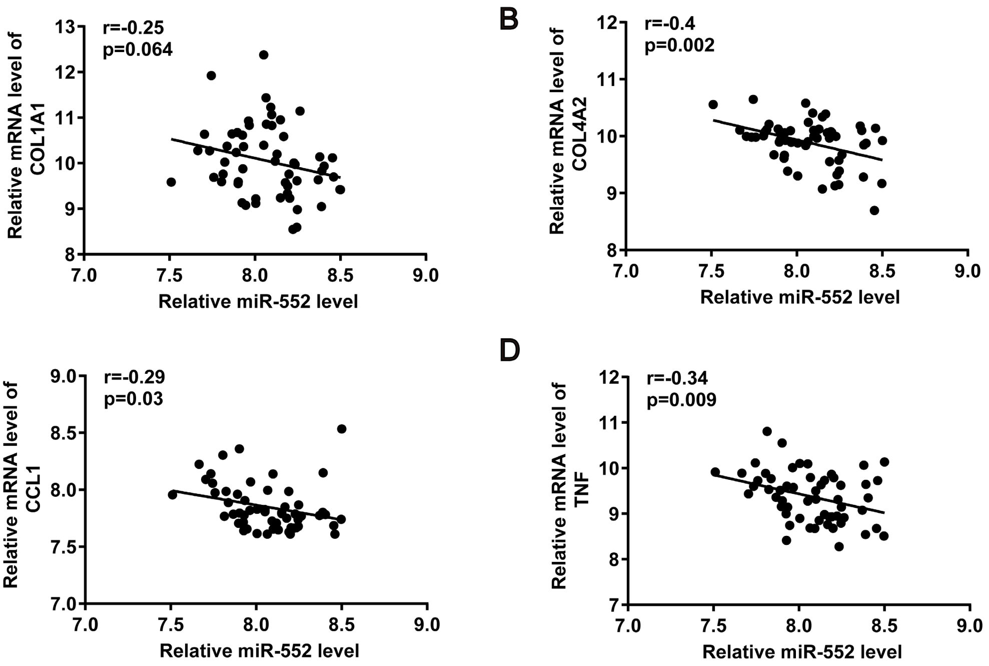

Figure S1 miR-552 is inversely correlated with fibrotic and inflammatory genes of MAFLD patients from GSE89632

(A) Correlation between miR-552 content and the mRNA levels of COL1A1 and COL4A2 in the liver of patients with MAFLD (n=39) and healthy controls (n=18). (B) Correlation between miR-552 content and the mRNA levels of CCL1 and TNF in the liver of patients with MAFLD (n=39) and healthy controls (n=18).

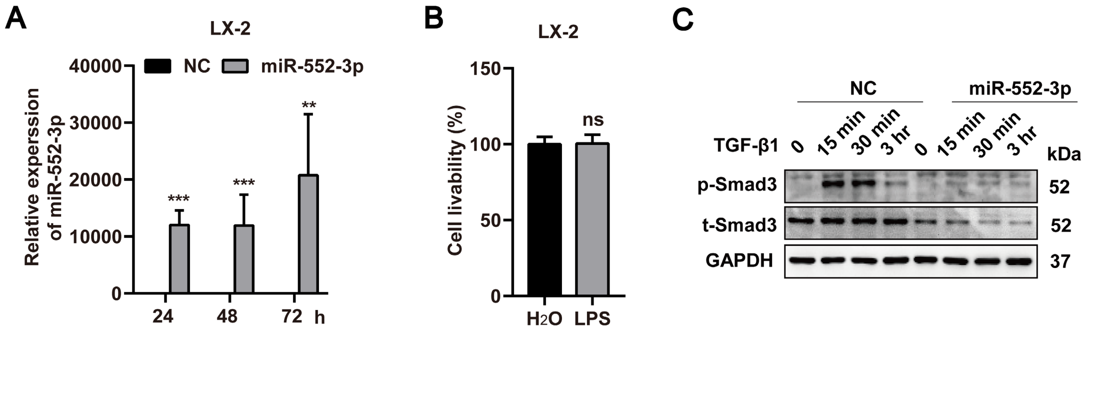

Figure S2 MiR-552-3p downregulates p-Smad3 in LX-2 cells after TGF-β1 treated for less than 3h

(A) Relative mRNA levels of miR-552-3p (1 nM) in LX-2 cells after transfected for 24, 48 and 72 h. (B)

The cell livability of LX-2 cells after treated with LPS (800 ng/ml) for 8h. (C) The protein levels of p-Smad3 and t-Smad3 in LX-2 cells after treated with TGF-β1 for 15 min, 30 min and 3 h. ** P<0.01, ***P<0.001 *vs*. NC; ns, non-significant.

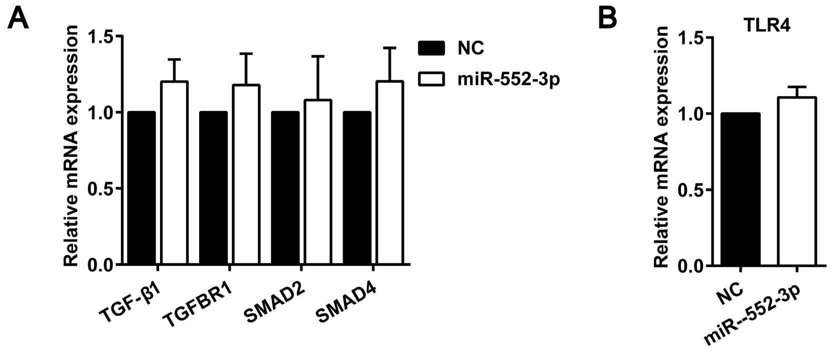

Figure S3 MiR-552-3p doesn't affect the expression of TGF-β1, TGFBR1, SMAD2, SMAD4 and TLR4 in LX-2 cells

(A) Relative mRNA levels of TGF-β1, TGFBR1, SMAD2 and SMAD4 of TGF-β1/Smad3 signaling pathway in LX-2 cells transfected with miR-552-3p (1 nM) for 48 h. (B) Relative mRNA level of TLR4 in LX-2 cells transfected with miR-552-3p (1 nM) for 48 h.

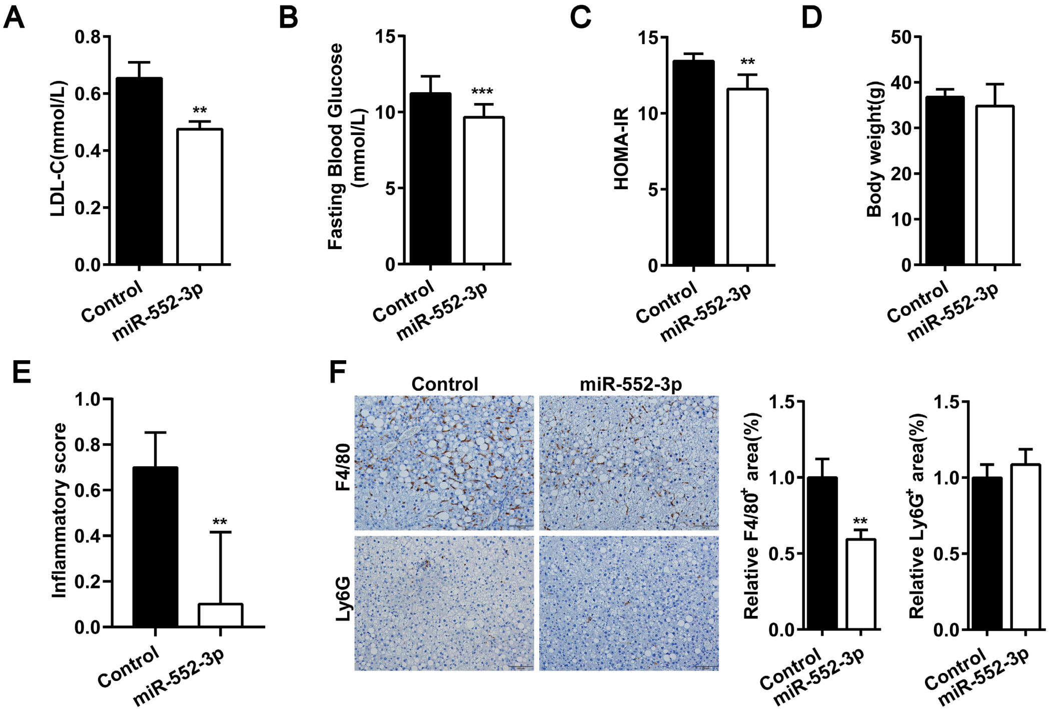

Figure S4 MiR-552-3p regulates glycolipid metabolism disorders in HFHFrHC diet-induced NASH mouse model

(A-C) The content of LDL-C in serum, fasting blood glucose and HOMA-IR index of HFHFrHC diet induced NASH mouse model. (D) The body weight of mice. (E) The inflammatory score of HFHFrHC diet induced NASH model with or without miR-552-3p. (F) The F4/80 and Ly6G staining of liver tissues in HFHFrHC diet induced NASH model. Data are presented as the mean ± SEM. n=10, ** P<0.01, ***P<0.001 *vs*. Control.

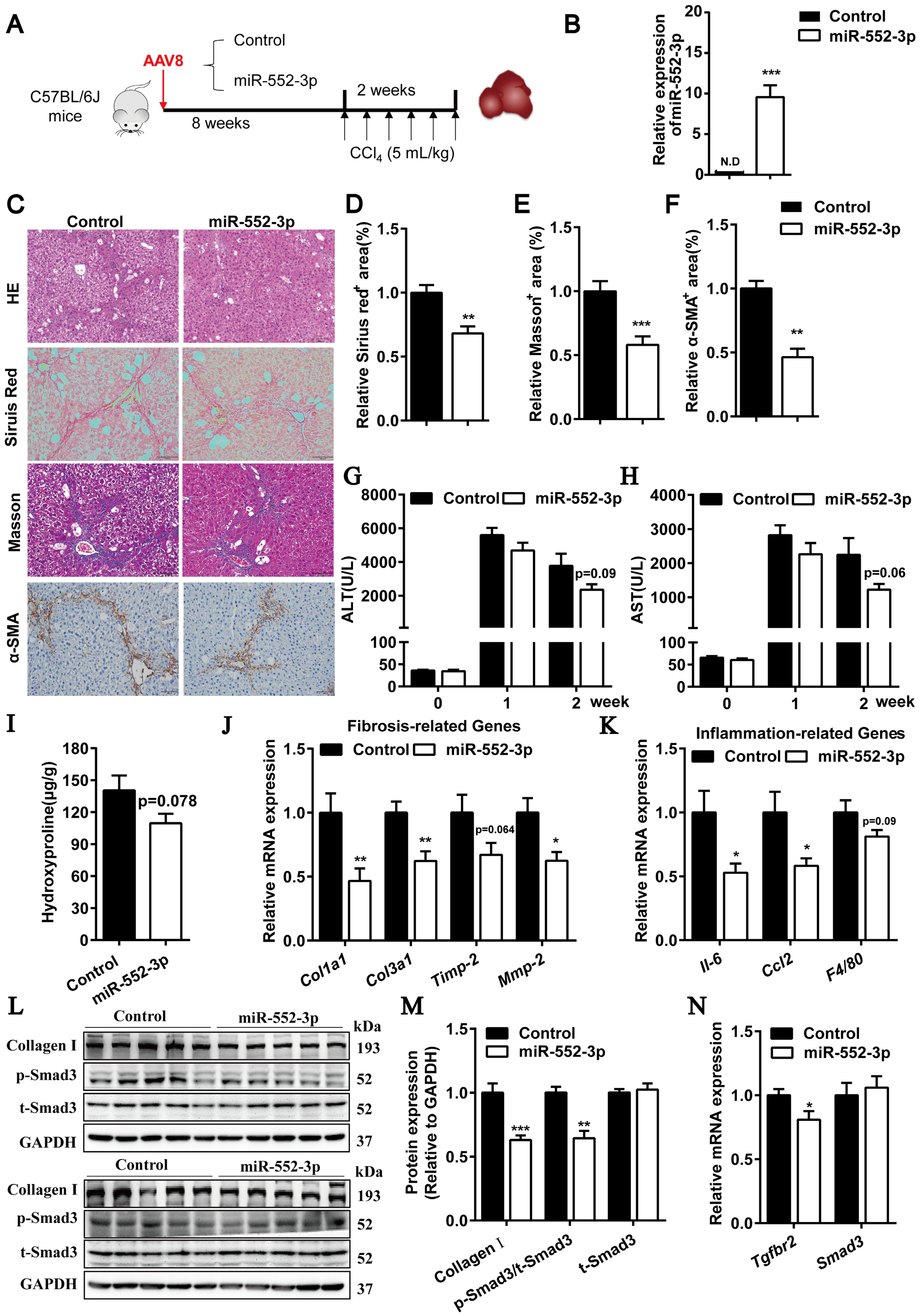

Figure S5 MiR-552-3p relieves the liver fibrosis and inflammation in CCl_4 induced animal model overexpressed by AAV8.

(A) Schematic diagram of CCl_4-induced animal experiment. (B) Relative level of miR-552-3p in the liver

tissues. (C) HE, Sirius red, Masson and α-SMA staining of liver samples. Scale bar: 100 μm. (D-F) The positive area of Sirius red, Masson and α-SMA staining. (G-H) ALT and AST levels in mouse serum tested every one week. (I) The content of hydroxyproline in mouse livers. (J-K) Relative mRNA levels of fibrotic and inflammatory genes in mouse livers. (L-M) Protein expression levels of Collagen I, p-Smad3 and t-Smad3 in mouse livers. (N) Relative mRNA levels of Tgfbr2 and Smad3 in mouse livers. Data are presented as the mean ± SEM. n=10, *P<0.05, ** P<0.01, ***P<0.001 *vs.* Control.

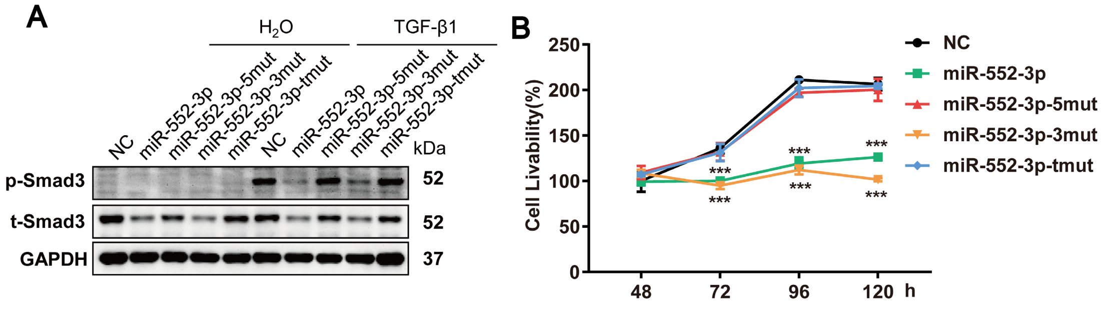

Figure S6 The inhibitory effect of miR-552-3p on the proliferation of LX-2 cells is dependent on its seed sequence

(A) The protein levels of p-Smad3 and t-Smad3 in the LX-2 cells after treated with TGF-β1 for 15 min.

(B) The cell livability of LX-2 cells transfected with NC, miR-552-3p, miR-552-3p-5mut, miR-552-3p-3mut or miR-552-3p-tmut (1 nM) for 48, 72, 96 and 120 h.

Table S1: Sequences of siRNAs and miRNAs

Name	Sequence
si-IPO8-1	UUAGUGAGAGUCCAAUUAAUT
si-IPO8-2	UGAGCUCAAUCUAAGAAAUUT
Anti-NC	UCACAACCUCCUAGAAAGAGUAGA
Anti-miR-552-3p	AACAGGUGACUGGUUAGACAA
Negative Control (NC)	UUCUCCGAACGUGUCACGUTT

Table S2: Information of real time PCR specific primers used in this study

Gene	Forward (5'→ 3')	Reverse (5'→ 3')
h-ACTA2	AAAAGACAGCTACGTGGGTGA	GCCATGTTCTATCGGGTACTTC
h-COL1A1	GCTTCACCTACAGCGTCACTGTCG	AGAGGAGTTTACAGGAAGCAGACAG
h-COL3A1	GGAGCTGGCTACTTCTCGC	GGGAACATCCTCCTTCAACAG
h-TIMP-2	AAGCGGTCAGTGAGAAGGAAG	GGGGCCGTGTAGATAAACTCTAT
h-MMP-2	TACAGGATCATTGGCTACACACC	GGTCACATCGCTCCAGACT
h-IL-6	ACTCACCTCTTCAGAACGAATTG	CCATCTTTGGAAGGTTCAGGTTG
h-CCL2	CAGCCAGATGCAATCAATGCC	TGGAATCCTGAACCCACTTCT
h-GAPDH	GGAGCGAGATCCCTCCAAAAT	GGCTGTTGTCATACTTCTCATGG
m-Col1a1	GCTCCTCTTAGGGGCCACT	ATTGGGGACCCTTAGGCCAT
m-Col3a1	CTGTAACATGGAAACTGGGGAAA	CCATAGCTGAACTGAAAACCACC
m-Timp-2	TCAGAGCCAAAGCAGTGAGC	GCCGTGTAGATAAACTCGATGTC
m-Mmp-2	ACCTGAACACTTTCTATGGCTG	CTTCCGCATGGTCTCGATG
m-Il-6	CTGCAAGAGACTTCCATCCAG	AGTGGTATAGACAGGTCTGTTGG
m-Ccl2	TAAAAACCTGGATCGGAACCAAA	GCATTAGCTTCAGATTTACGGGT
m-Gapdh	AGGTCGGTGTGAACGGATTTG	GGGGTCGTTGATGGCAACA
